# Insights into the Metabolic Response of *Lactiplantibacillus plantarum* CCFM1287 upon Patulin Exposure

**DOI:** 10.3390/ijms231911652

**Published:** 2022-10-01

**Authors:** Chaozhi Wei, Chuan Zhang, Yuhang Gao, Leilei Yu, Jianxin Zhao, Hao Zhang, Wei Chen, Fengwei Tian

**Affiliations:** 1 State Key Laboratory of Food Science and Technology, Jiangnan University, Wuxi 214122, China; 2 School of Food Science and Technology, Jiangnan University, Wuxi 214122, China; 3 National Engineering Research Center for Functional Food, Jiangnan University, Wuxi 214122, China

**Keywords:** *Lactiplantibacillus*, patulin, toxicity, oxidative stress, metabolome, purine metabolism

## Abstract

Patulin (PAT) is a common mycotoxin in the food industry, and is found in apple products in particular. Consumption of food or feed contaminated with PAT can cause acute or chronic toxicity in humans and animals. *Lactiplantibacillus plantarum* CCFM1287 is a probiotic strain that effectively degrades PAT in PBS and food systems. In this study, it was found that the concentration of PAT (50 mg/L) in MRS medium decreased by 85.09% during the first stages of CCFM1287 growth, and this change was consistent with the first-order degradation kinetic model. Meanwhile, the regulation of oxidative stress by *L. plantarum* CCFM1287 in response to PAT exposure and metabolic changes that occur during PAT degradation were investigated. The degree of intracellular damage was attenuated after 16 h of exposure compared to 8 h. Meanwhile, metabolomic data showed that 30 and 29 significantly different metabolites were screened intracellularly in the strain after 8 h and 16 h of PAT stress at 50 mg/L, respectively. The results of pathway enrichment analysis suggested that the purine metabolic pathway was significantly enriched at both 8 h and 16 h. However, as is consistent with the performance of the antioxidant system, the changes in *Lactiplantibacillus* diminished with increasing time of PAT exposure. Therefore, this study helps to further explain the mechanism of PAT degradation by *L. plantarum* CCFM1287.

## 1. Introduction

Patulin (PAT) is a toxic secondary metabolite produced by *Penicillium*, *Aspergillus*, and other fungi and is widely found in fruits, especially apples and apple products. After PAT is ingested, it induces oxidative stress in the body as well as, causes gastrointestinal disturbances with vomiting and nausea, and disordered edema, and tissue damage to blood-rich organs, such as the liver and kidneys [1,2,3]. Owing to its inherent stability in thermal and acidic environments, PAT can withstand adverse conditions during food processing. Therefore, commercial food products can pose a risk of exposure, which can cause potential harm to consumers [4,5,6]. Hence, the reduction of PAT in food products is a global safety issue that needs to be emphasized to ensure human health.

Research on PAT reduction has increased in recent years. Compared to physical and chemical methods, the biological control method of PAT has a higher degradation efficiency and is more environmentally friendly [1]. *Pichia guilliermondii* [7,8], *Pichia caribbica* [9], *Pseudomonas aeruginosa* [10], and *Lactiplantibacillus* [11,12] exhibit the ability to degrade PAT, although their degradation products differ. In our previous study, it was found that *L. plantarum* CCFM1287 could degrade PAT to E-ascladiol in PBS and apple juice [13]. Compared to other species, *L. plantarum* CCFM1287 is a classical and typical probiotic that can be used not only to remove PAT from the system but also to alleviate the toxic effects caused by PAT through its probiotic properties. In addition, as a strain with proven safety, *L. plantarum* can be added directly to food [14,15]. Therefore, further investigations need to be conducted based on these results. 

Current research on PAT biodegradation is mostly focused on the exploration of degradation strains, optimization of reaction systems, and discussion of degradation mechanisms [7,10,11,16]. Several recent studies have contributed to the speculation of the degradation pathway of PAT and screening of intracellular degradation enzymes. However, there remains a lack of research on whether the presence of PAT affects the metabolism of degrading strains. Additionally, PAT exposure causes oxidative stress, DNA damage, and apoptosis in cells and tissues [4]. How PAT stress damages the degrading strain *L. plantarum* CCFM1287 is another research gap worth exploring. He et al. [17] investigated the inhibitory effect of genistein on citrinin production by *Monascus aurantiacus* in 2020. Qiu et al. [18] analyzed the metabolic changes of *Aspergillus niger* FS10 under AFB1 stress in the next year. These studies have aided in clarifying the mechanisms by which microorganisms inhibit toxin synthesis or degrade toxins. Therefore, the results of this study are expected to further explain the degradation mechanism of PAT and guide future applications of the PAT-degrading *L. plantarum* CCFM1287 strain.

Our previous research illustrated that *L. plantarum* CCFM1287, isolated from Chinese traditional fermented foods, is a probiotic that degrades PAT in vitro and has potential beneficial effects in vivo [13]. Hence, the aims of this study were to: (1) clarify the effect of PAT on oxidative stress in *L. plantarum* CCFM1287 at different times, (2) analyze the changes in the strain at the metabolic level under PAT stress, and (3) reveal the mechanism of PAT stress interference with the metabolic network at the phylogenetic level of *L. plantarum* CCFM1287.

## 2. Results

### 2.1. Effect of Different Concentrations of PAT on L. plantarum CCFM1287 Cell Growth

**Figure 1:**
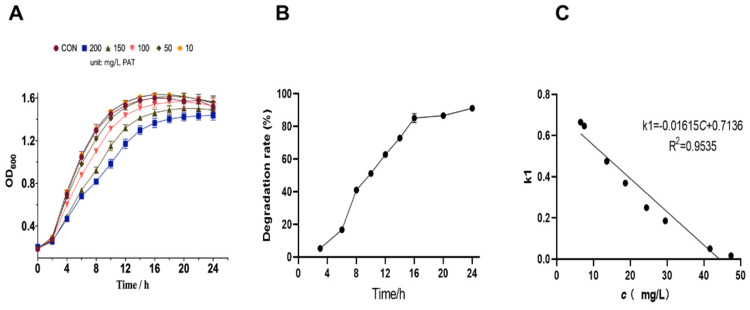
Growth curve of *Lactiplantibacillus plantarum* CCFM1287 under PAT stress (**A**). Biodegradation of PAT by *L. plantarum* CCFM1287 in MRS medium (**B**). Kinetic fitting results (**C**).

The growth of *L. plantarum* CCFM1287 under different concentrations of PAT is shown in Figure 1A. The inhibitory effect on *L. plantarum* CCFM1287 was more obvious as the PAT concentration increased. The time taken for *Lactiplantibacillus* treated with 200 mg/L PAT to enter the logarithmic growth phase was delayed by 2 h compared to that in the control group. Meanwhile, it was found that the growth curves of the control group and the 10 and 50 mg/L PAT treatment groups were similar, with a lot of overlap. Therefore, it was speculated that *L. plantarum* CCFM1287 exhibited significant tolerance to 50 mg/L PAT treatment; therefore, this concentration was selected for subsequent experiments. In a previous study, *L. plantarum* CCFM1287 was found to degrade PAT to *E*-ascladiol in PBS [13]. In this study, the PAT degradation capacity of *L. plantarum* CCFM1287 was measured in liquid medium. In MRS medium with a PAT concentration of 50 mg/L, *L. plantarum* CCFM1287 initially showed good PAT degradation, with the steepest slope of the degradation curve 6–8 h and a moderate adjustment of the degradation rate occurring between 16 and 24 h. After 24 h, the PAT degradation rate reached 92.77%. Combining the growth curves under PAT stress, the metabolic changes of the degrading strains under PAT stress were studied at 8 h and 16 h.

In addition, the degradation process of PAT during the growth of CCFM1287 in the medium containing PAT was in accordance with the first-order degradation kinetic model. The first-order reaction kinetics can be expressed by using

$$\mathrm{In}\frac{C0}{\mathrm{Ct}}=\mathrm{kt}$$

where C0 is the concentrations of PAT in the initial state, Ct is the concentrations of PAT in the stable state, k is the first-order kinetic constant, and t is the reaction time.

Figure 1B shows that the change in PAT concentration reached stability when the reaction reached 20 h. During the first 20 h of CCFM1287 growth, the PAT concentration changed from 47.34941 mg/L to 6.66713 mg/L, and the first-order kinetic constant (k) ranged from 0.66490 to 0.01618. Thus, the function between the constant k and the PAT concentration *C* was as follows:

k = −0.01615*C* + 0.7136.

After fitting, R^2^ = 0.9535 > 0.9, the degradation curve of PAT was consistent with the first-order kinetic model [8,19].

### 2.2. Changes in Total Intracellular Antioxidant Capacity and Superoxide Dismutase Activity Triggered by PAT 

**Figure 2:**
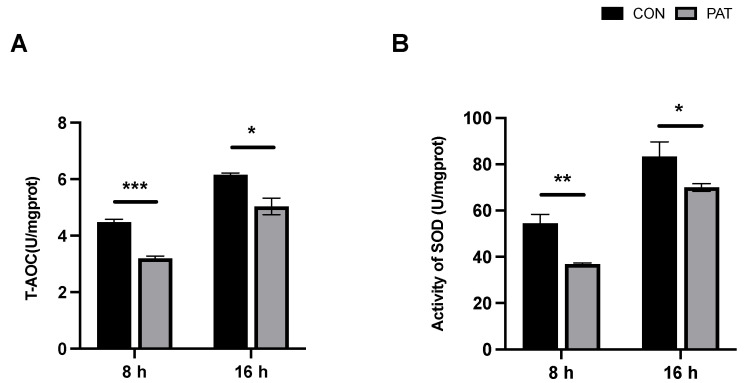
Total intracellular antioxidant capacity (T-AOC) (**A**) and superoxide dismutase (SOD) activity (**B**) of *L. plantarum* CCFM1287 exposed to different reaction times. (* *p* < 0.05, ** *p* < 0.01, *** *p* < 0.001 vs. control group).

The total intracellular antioxidant capacity (T-AOC) is an indicator of the integrated state of bacterial cells. Throughout the response period, the T-AOC increased with time in both the control and PAT-stress groups (Figure 2A). However, a significant downregulation of T-AOC occurred in the PAT−stressed groups compared to that in the control group at 8 h and 16 h. Moreover, this downregulation was more pronounced at 8 h than at 16 h. A similar trend was observed in the superoxide dismutase (SOD) activity assay. As a typical antioxidant enzyme, SOD is considered the first line of defense against oxidative stress, and its activity largely determines the resistance of *lactiplantibacillus* to oxidative damage. Figure 2B shows that SOD activity increased with the growth of *L. plantarum* CCFM1287. However, under PAT stress, SOD activities at different reaction times significantly decreased compared to those in the control group, and the changes were more significant in the 8 h group.

### 2.3. Changes in Metabolic Capacity Triggered by PAT

**Figure 3:**
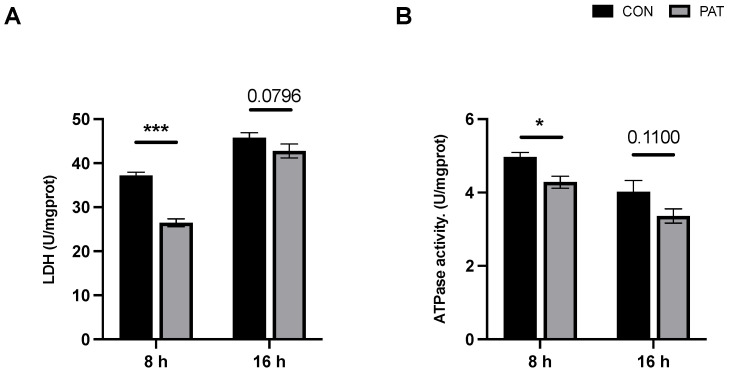
The lactate dehydrogenase (LDH) activity (**A**) and Na^+^–K^+^ ATPase activity (**B**) of *L. plantarum* CCFM1287 exposed to different reaction times. (* *p* < 0.05, *** *p* < 0.001 vs. control group).

Lactate dehydrogenase (LDH) is a key enzyme in the metabolism of *lactiplantibacillus* and is also a potential target indicator of *lactiplantibacillus* under the stress of harmful factors. Figure 3A indicates that the intracellular LDH activity of *L. plantarum* CCFM1287 was downregulated under PAT stress, but this downregulation was not significant after 16 h (*p* = 0.0796). Na^+^–K^+^ ATPase is a transmembrane enzyme that plays a crucial role in osmotic pressure regulation and energy metabolism and is a bioindicator of *lactiplantibacillus* activity. Unlike other indicators, ATPase showed an overall decreasing trend as the growth time of the strain increased (Figure 3B). Nevertheless, PAT stress still modulated the ATPase activity of *L. plantarum* CCFM1287, and similar to LDH activity, the change was not significant in the 16 h group (*p* = 0.110).

### 2.4. Changes in Metabolic Capacity Triggered by PAT

**Figure 4:**
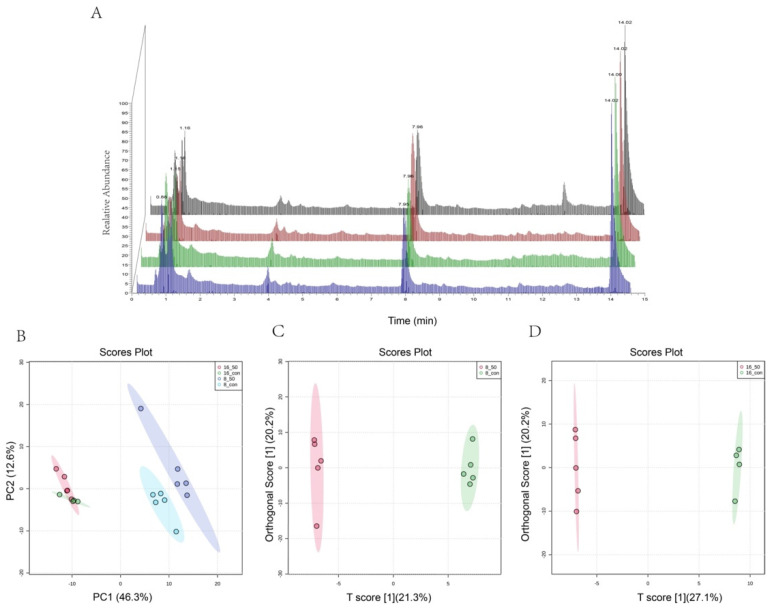
Comparative analysis of intracellular metabolites in *L. plantarum* CCFM1287 during PAT exposure. Total ionogram (TIC) plots for each treatment group (**A**). Control group 8 h (red), PAT group 8 h (blank), control group 16 h (green), and PAT group 16 h (blue). PCA of the PAT exposure and control groups in the 8 and 16 h treatment groups (**B**). The OPLS-DA of the PAT-exposure and control groups with 8 h of reaction time (**C**), and in the 16 h of reaction time (**D**). Results are shown as the mean of four or five replicates.

To verify that *L. plantarum* CCFM1287 underwent changes after PAT exposure, its intracellular metabolic profile was compared. As displayed in Figure 4A, *L. plantarum* CCFM1287 showed little difference in the total metabolites after PAT stress, but differences were found in the total ionogram (TIC) at 0.88, 3.99, 7.95, and 12.44 min, suggesting that PAT exposure causes *L. plantarum* CCFM1287 to change at the metabolite level, which is in accordance with the results of the previous phase of this research. From the results shown in Figure 4B, it is clear that the time of strain growth has a significant effect on the alteration of intracellular metabolism. There was an obvious clear separation between the control and PAT exposure groups with 8 and 16 h reaction times. The orthogonal projections to latent structures discriminant analysis (OPLS-DA) model was used to analyze the dispersion of the samples and the two reactions (Figure 4C,D). The T1 scores of 21.3% and 27.1% in Figure 4C,D, respectively, imply a significant effect of PAT exposure on *L. plantarum* CCFM1287 intracellular metabolism at both the 8 and 16 h treatment time points. The results were analyzed by using a permutation test to verify that the models were not overfitted. The prediction accuracy (Q^2^) of both models was higher than 0.8, which indicated that the models were not overfitted and had good predictive ability.

### 2.5. PAT Exposure Triggers Changes in Intracellular Metabolites of L. plantarum

**Figure 5:**
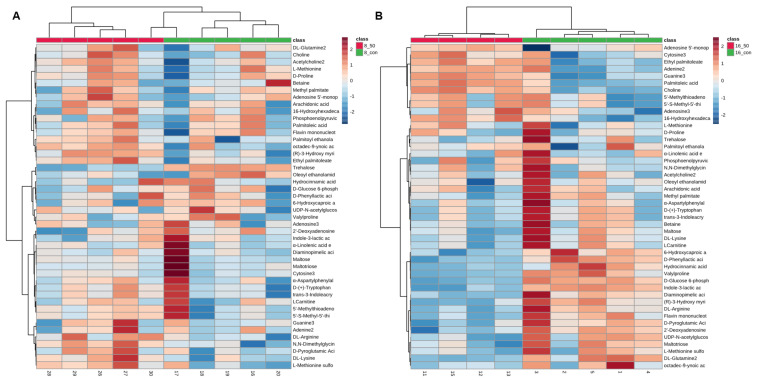
Heat map of endogenous differential metabolites based on PAT exposure. The 8 h group (**A**) and the 16 h group (**B**). These data are conditioned by *p* < 0.05, fold change (FC) > 1.2, and projection value (VIP) > 1 screening criteria.

Subsequently, 198 intracellular metabolites of *L. plantarum* CCFM1287 were identified by using C. D. 3.2 software (Thermo Scientific, Boston, MA, USA). Significantly different metabolites were determined by using the module on the online analysis website MetaboAnalyst 5.0 (https://www.metaboanalyst.ca) (accessed on 1 July 2022), identifying *p* < 0.05, fold change (FC) > 1.2, and projection value (VIP) > 1 as the screening criteria. At 8 h, there were 30 significantly different metabolites in *L. plantarum* CCFM1287, whereas 28 significantly different metabolites were screened at 16 h, including 15 categories of carbohydrates, fatty acids, amino acids, nucleotides, and organic acids. To better show the changes in metabolites under PAT stress, the significantly different metabolites were selected for hierarchical Pearson clustering, and the results showed significant differences between the control and PAT treatment groups, which was consistent with the results of the PCA and OPLS-DA models (Figure 5). Meanwhile, in both treatment groups, maltotriose, ethyl palmitoleate, α-linolenic acid ethyl ester, L-glutamine, dimethylglycine, valylproline, diaminopimelic acid, D-pyroglutamic acid, adenosine 5′-monophosphate, α-asparagylphenylalanine, and guanine showed significant changes in metabolic levels (*p* < 0.05).

### 2.6. PAT Exposure Affects Intracellular Metabolic Pathways in L. plantarum

**Figure 6:**
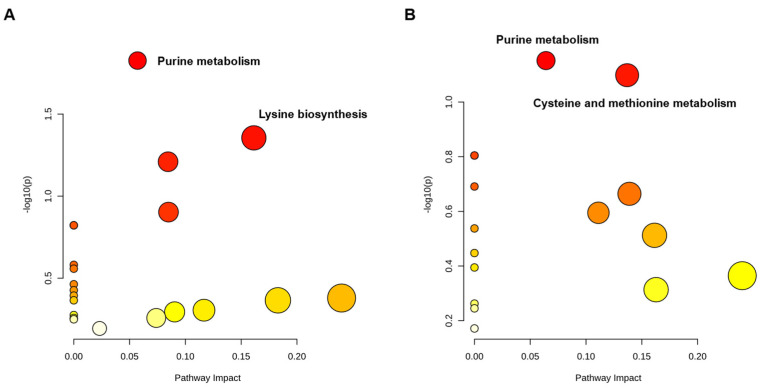
Enrichment diagram of the *L. plantarum* metabolic pathway after PAT exposure. (**A**) The 8 h group and (**B**) the 16 h group.

A pathway enrichment analysis was performed for each subgroup of metabolites to further investigate the effect of PAT exposure on *L. plantarum* CCFM1287 metabolism. The effects of each metabolic pathway are plotted as horizontal coordinates, and the abundance of metabolic pathways is plotted as the vertical coordinates. The larger the area of dots in Figure 6, the greater the impact of the pathway on *L. plantarum* CCFM1287 metabolism, and a darker color indicates a higher abundance of pathway enrichment.

As shown in Figure 6A, purine metabolism was the prominent pathway subjected to PAT stress after 8 h of toxin exposure (*p* = 0.014), whereas the lysine biosynthesis pathway was also enriched (*p* = 0.044). However, after 16 h of exposure, the purine metabolic pathway remained prominent in the intracellular metabolism, but a significant difference was not observed (*p* = 0.070). The same was observed for cysteine and methionine metabolic pathways (*p* = 0.079). Notably, based on the decrease in purine metabolism with time, it was hypothesized that the metabolism of *L. plantarum* CCFM1287 is influenced by PAT, decreases with time, and shows a tendency to be tolerated.

**Figure 7:**
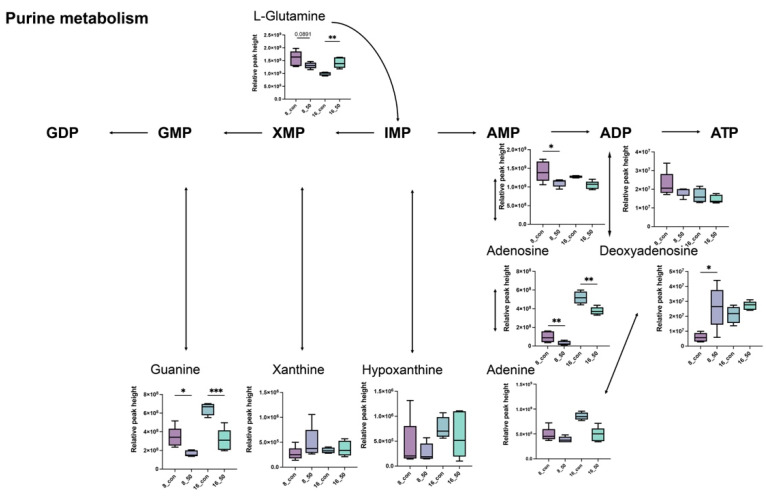
Intracellular purine metabolism pathway of *L. plantarum* CCFM1287 under (partial) PAT exposure. (* *p* < 0.05, ** *p* < 0.01, *** *p* < 0.001 vs. control group).

Changes in the intracellular metabolites of *L. plantarum* CCFM1287 following PAT exposure were further explored. As shown in Figure 6 and Figure 7, the intracellular purine metabolic pathway was significantly enriched following PAT exposure. Mass spectrometry was used to determine the relative abundances of L−glutamine, guanine, AMP, adenosine, adenine, and deoxyadenosine, which showed significant differences (*p* < 0.05). Moreover, the abundance of all detected metabolites in metabolome, except deoxyadenosine, in the purine metabolic pathway showed a decreasing trend after PAT exposure. Cysteine and methionine metabolisms and starch and sucrose metabolisms also exhibited a trend of enrichment after PAT exposure.

**Figure 8:**
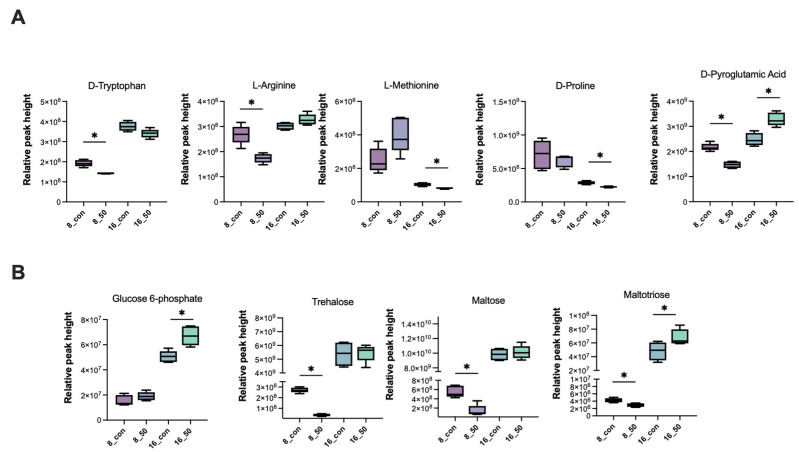
Changes in selected differential metabolites, including (**A**) amino acids and (**B**) carbohydrates. (* *p* < 0.05 vs. control group).

In addition, certain amino acid levels changed significantly after PAT exposure. Tryptophan and arginine were significantly downregulated at 8 h, whereas methionine and proline were inhibited at 16 h. However, the change in pyroglutamic acid was significant at 8 and 16 h, although it was inhibited at 8 h and then promoted (Figure 8A). Among the changes in carbohydrates, we found that trehalose and maltose were significantly inhibited at 8 h, whereas at 16 h the difference in the changes caused by PAT exposure was not significant, despite the increase in the intracellular metabolite concentration. Glucose 6-phosphate, a key metabolite in energy metabolism, was also significantly promoted at 16 h. Another metabolite, maltotriose, which was significant at different time points, showed similar trends in its levels to those of pyroglutamic acid.

## 3. Discussion

Because PAT is a mycotoxin commonly found in apples and products made from apples, it is necessary to investigate the use of biological methods to control it. In a previous study, we screened a strain of *L. plantarum* CCFM1287 that can degrade PAT to E−ascladiol in its resting state in PBS and explored its degradation mechanism [9]. However, there is a lack of studies on the effect of PAT exposure on degradation strains. Therefore, to further analyze the mechanism of PAT degradation by *L. plantarum* CCFM1287, the effect of PAT exposure on intracellular indicators and the metabolism of *Lactiplantibacillus* was analyzed in this study. 

Growth curves were generated for *L. plantarum* CCFM1287 cells in MRS medium with or without different doses of PAT treatment to determine the PAT dose and response time points. To ensure that the cells in the control and PAT-exposed groups were in the same physiological state, a PAT concentration of 50 mg/L, at which point PAT is inhibitory but not lethal to the cells, was chosen for subsequent experiments. These results were similar to the findings of Horváth et al. [20] (published in 2010) and Iwahashi et al. (published in 2006) [21]. Meanwhile, the strain was inoculated in MRS medium containing PAT, and the residual amounts of PAT were detected at different time points. The results indicated that the highest slope of PAT clearance was between 6 h and 16 h. However, in combination with the growth conditions of the strain, 8 h and 16 h were chosen as the experimental time points. Notably, *E*-ascladiol, a degradation metabolite of PAT, was also identified in the medium, and it increased with time. Meanwhile, the trend of PAT degradation can be well fitted with the first-order degradation kinetic model. This further emphasizes the role of *L. plantarum* CCFM1287 in PAT degradation. 

PAT causes the dysregulation of the intracellular antioxidant system via reactive oxygen species (ROS)—mediated endoplasmic reticulum stress, which in turn causes oxidative damage and cell apoptosis [22,23]. SOD is the main scavenger of ROS and is involved in antioxidant defense, where the total antioxidant capacity (T-AOC) can reflect the total ability of the cell to scavenge ROS to some extent. Significant inhibition of SOD and T-AOC was observed at 8 h and 16 h in the PAT—exposed group, suggesting PAT—induced oxidative stress in the cells and that the oxidative damage was sustained. Interestingly, the oxidative damage to cells at 16 h was weaker than that at 8 h of PAT exposure. Similar results were observed by two other studies, wherein *Pediococcus pentosaceus* R1 resisted oxidative stress and *A. niger* FS10 antagonized AFB1 [18,24]. The strains were affected by deleterious factors, which decreased over time and showed gradual recovery. Moreover, LDH is a key enzyme for the growth and metabolism of *L. plantarum*, which can reflect the viability of the bacteria [25,26,27]. Na^+^–K^+^ ATPase is a specific protein found in cell membranes that not only maintains transmembrane transport and osmotic pressure homeostasis but is also involved in various protein interactions and the regulation of signaling, such as the activation of mitogen−activated protein kinases (MAPK) and ROS production [27]. Both LDH and ATPase are important indicators of cellular metabolic activity. In the present study, we hypothesized that the downregulation of LDH and ATPase upon PAT exposure was closely related to oxidative stress. Excess ROS formed by PAT stress downregulates LDH activity, which in turn inhibits cell growth and viability. This phenomenon was also verified in a study by Zhang et al. in 2016, in which LDH activity was significantly inhibited in *Escherichia coli* during heat stress [28]. Furthermore, LDH inactivation and inhibition lead to increased leakage of proteins and other macromolecules, further affecting intracellular homeostasis. Additionally, the significant inhibition of Na^+^–K^+^ ATPase may be caused by a series of responses resulting from PAT exposure. First, PAT stress induces excessive intracellular ROS production, leading to the dysregulation of antioxidant systems, such as SOD enzyme activity, as well as an increase in malondialdehyde content, which can alter cell membrane permeability, integrity, and depolarization, and ultimately cause a decrease in ATPase activity [29,30]. Similar to the antioxidant system response, the inhibition of LDH and ATPase also diminished with increasing PAT exposure time. Compared with 8 h, the inhibition at 16 h was no longer significant. In summary, PAT exposure causes damage to the intracellular antioxidant system of *L. plantarum* CCFM1287 and affects the activity of some metabolic marker enzymes, but these negative effects diminish over time.

**Figure 9:**
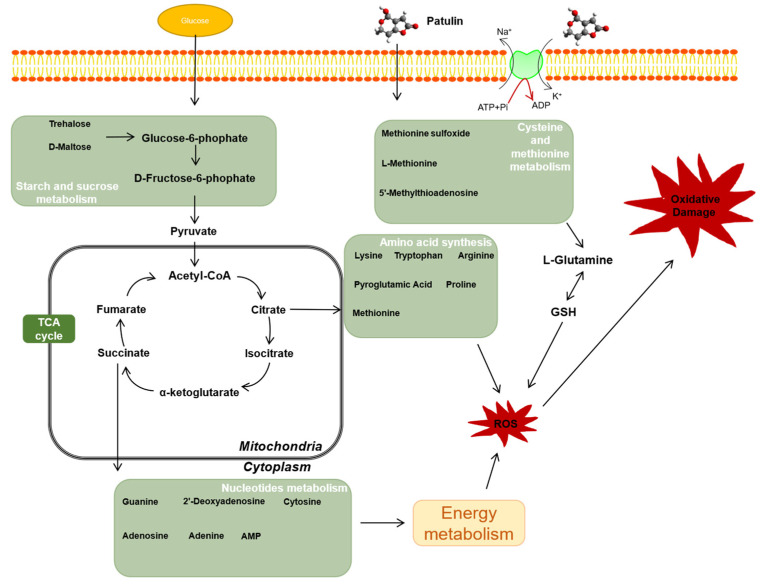
Model for the response of *L. plantarum* CCFM1287 to PAT exposure using the metabolome.

Metabolites are a class of small-molecule compounds with weights below 1000 kDa that are produced at all stages of life. In toxicological studies, changes in metabolites are key factors that reflect cellular responses to negative stress [31]. As a new technology, metabolomic analysis can target core biomarkers that are more sensitive than phenotypic indicators. As shown in Figure 9, PAT exposure not only altered *L. plantarum* CCFM1287 antioxidant and metabolic viability indicators, but also led to changes in metabolites and metabolic pathways. Purine metabolism is the most significant metabolic pathway enriched after PAT exposure. Purines are important components of nucleotides and are essential for cell structure, energy metabolism, and other functions. The levels of guanine, adenine, and adenosine were significantly decreased in the PAT—treatment group, which may have been due to a decrease in DNA and RNA biosynthesis. As a biomarker after DNA-framework attack, the abundance of deoxyadenosine was significantly upregulated, which is the same phenomenon observed by Xu et al. [31] in *E. coli* (published 2021). ATP is an important product of purine metabolism, and it is considered the most efficient energy source for physiological reactions. Combined with a reduction in ATPase activity, exposure to PAT is thought to negatively affect the normal energy metabolism of *L. plantarum* CCFM1287. However, purines can be used as key additives in some bacterial cultures as nitrogen sources and substrates, and significantly inhibited guanine and adenine can be selected as potential metabolites to increase the energy metabolism level of the strain and enhance tolerance to PAT. Meanwhile, the metabolite 5′−methylthioadenosine in the cysteine and methionine metabolic pathways, which is used as a source of purines and methionine in microorganisms, was also significantly upregulated by PAT treatment. This further emphasizes the status of purine metabolism after PAT exposure [32]. In general, purines exhibit diverse function in cellular metabolism. Not only do purines provide materials for nucleic acid synthesis, but they are also involved in signaling pathway and energy metabolism [33]. The lower purine abundance of CCFM1287 under PAT exposure reflects less energy production and inhibition of cellular metabolism, which corresponds to the results of the previous experiments on antioxidant capacity and metabolic enzymes, further illustrating the negative effect of PAT on the degrading strain.

Amino acids play an important role in energy metabolism and antagonize harmful exogenous factors within *Lactiplantibacillus* [34]. However, amino acids are expressed at different levels during PAT exposure. In addition, amino acid act as precursors for energy generation by gluconeogenesis, and changes in amino acid expression levels also indicate fluctuations in energy metabolism following PAT exposure [35,36]. Levels of L-tryptophan with its antioxidant activity and L-pyroglutamate, a downstream metabolite of glutathione, were decreased compared with those of the control group, which may be related to antioxidant depletion under oxidative stress. Downregulation of arginine and lysine metabolism under PAT exposure leads to the inhibition of the synthesis of specific functional proteins, which further exacerbates the metabolic disorder in *Lactobacillaceae*. In addition, after 16 h of PAT exposure, the increase in L−glutamine may not only be due to the preparation for synthesis of the antioxidant glutathione, but may also be an important node of intracellular carbon and nitrogen metabolism and enhance the energy metabolism of the strain [37].

Carbohydrate metabolism also plays an important role in the energy metabolism of *L. plantarum* CCFM1287. Glucose-6-phosphate, which is a marker metabolite generated after glucose phosphorylation and is involved in biochemical pathways such as the pentose phosphate pathway and glycolysis, was significantly upregulated after PAT exposure [38]. Therefore, it was hypothesized that the energy supply system in purine metabolism is inhibited under PAT stress, which in turn stimulates the carbohydrate metabolism. Meanwhile, the levels of trehalose and maltose, which are upstream raw materials in the starch and sucrose metabolic pathways, decreased after PAT stimulation, with significant differences at 8 h, whereas the differences at 16 h were not significant. This further verified that the effect of PAT exposure on *L. plantarum* CCFM1287 diminished with increasing reaction time.

## 4. Materials and Methods

### 4.1. Chemicals and Bacterial Strain

Patulin (purity ≥ 99%) was purchased from Pribolab Ltd. (Qingdao, China) and used as the standard for subsequent experiments. The high analytical grade chemicals used for chromatography and mass spectrometry were purchased from Merck (Darmstadt, Germany).

*Lactiplantibacillus plantarum* (formerly *Lactobacillus plantarum* [39]) CCFM1287 (formerly *L. plantarum* 13M5) was obtained from the culture collection of Food Microbiology at Jiangnan University (Wuxi, China) and stored frozen at −80 °C in glycerol stock. The strain was reactivated by two passages on MRS agar plates prior to experimentation. 

### 4.2. Culture of L. plantarum CCFM1287 and Detection of PAT

*L. plantarum* CCFM1287 was inoculated with MRS liquid medium to prepare a 2% (*v*/*v*) inoculum and was incubated at 37 °C. MRS medium containing PAT under similar conditions, but without *Lactiplantibacillus* inoculation, was used as a control. The PAT concentration in the medium was measured every 2 h. Extraction was performed twice by using ethyl acetate. The extracts were combined and the eluate was evaporated by using a vacuum freeze dryer. Finally, the residue was redissolved in a methanol–water (1:9, *v*/*v*) solution. Subsequently, the extracts were passed through an organic microporous membrane (0.22 μm) and then collected in a feed vial for analysis by high-performance liquid chromatography with UV detection.

### 4.3. Determination of Important Intracellular Antioxidant and Physiological Indicators of Bacteria

The intracellular superoxide dismutase (SOD) and lactate dehydrogenase (LDH) levels, ATPase activities, and antioxidant capacity (T-AOC) of bacteria were measured separately for different PAT treatment times by using the relevant kits (Jiancheng Bioengineering Institute, China). These indicators were measured according to the manufacturer’s instructions. Briefly, the bacteria were collected and washed with PBS buffer, the cells were disrupted by an ultrasonic disruptor, the supernatant was collected by centrifugation and set aside, and the protein concentration was determined by using a BCA kit (Beyotime Institute of Biotechnology, China).

### 4.4. Sample Preparation for Metabolic Analysis

The sample preparation protocol was modified based on previous studies conducted in our laboratory [40]. Pre-PAT-treated *L. plantarum* was collected, the samples were placed on ice, and all subsequent experiments were performed at 4 °C. The sediment was treated with quenching solvent (20% MeOH/0.9% NaCl) for 10 min, and the supernatant was removed by centrifugation, followed by washing of the cell precipitate twice with washing solution (quenching solvent, 0.9% (*w*/*v*) NaCl in a 4:1 ratio). After sample quenching, metabolites were extracted by using 3 mL of extractant. After three freeze–thaw cycles (liquid nitrogen for 3 min and −20 °C for 20 min), the supernatant was collected by centrifugation. The resulting supernatants were combined and concentrated until dry in a vacuum concentrator (Thermo Fisher Scientific, Madison, WI, USA). Finally, 200 μL of the solution was re-solubilized with methanolic water (9:1).

### 4.5. Metabolite Analysis

Nontargeted metabolites in *Lactiplantibacillus* samples exposed to PAT were identified by using the method from Xu and Zhu et al. [41,42]. Their research will be published in 2021 and 2022, respectively. After metabolite extraction from *L. plantarum* CCFM1287, the metabolites were separated on a HSS T3 column (1.8 μm, 2.1 × 100 mm) (Waters, Boston, MA, USA) and analyzed by ultra-high-performance liquid chromatography (UPLC) with a Q Exactive mass spectrometer (Thermo Scientific, Boston, MA, USA). Following UPLC-Q-Exactive analysis, the raw data were imported into Compound Discover 3.2 (Thermo Scientific, Boston, MA, USA) for peak detection and alignment. To further improve differentiation between groups and screen for biomarkers, the online platform MetaboAnalyst 5.0 was used to perform PCA clustering analysis and orthogonal partial least squares discriminant analysis (OPLS-DA) on the metabolomic data. VIP values ≥ 1, fold change (FC) > 1.2, and projection value (VIP) > 1 were used to identify differential metabolites. Differential metabolites between groups were summarized and analyzed by using metabolic enrichment and pathway analysis based on a database search (KEGG, http://www.genome.jp/kegg/) (accessed on 10 July 2022).

### 4.6. Statistical Analysis

All experiments were performed independently, and intracellular data are expressed as the mean ± standard error of three parallel tests, whereas metabolomic analysis was performed for five parallel tests. SPSS v. 22 (Chicago, IL, USA) software was used for statistical analysis, and statistical significance was set at *p* < 0.05 by independent one-way ANOVA tests. Figures were plotted by using GraphPad Prism (San Diego, CA, USA).

## 5. Conclusions

In the present study, *L. plantarum* CCFM1287 was found to not only degrade PAT in the resting state, but also to have similar efficacy in normal growth metabolism. After PAT exposure, the oxidative stress capacity and metabolic levels of *L. plantarum* CCFM1287 were downregulated, but the strain could recover its metabolism over time. In addition, metabolomic analysis determined that purine, amino acid, and carbohydrate metabolisms were disturbed after PAT exposure. In conclusion, this study contributes to the understanding of the effect of PAT addition on the metabolism of degradation strains and may provide recommendations for the reduction of PAT contamination in food and feed products. 

## Data Availability

Not applicable.
